# Emerging role of substance and energy metabolism associated with neuroendocrine regulation in tumor cells

**DOI:** 10.3389/fendo.2023.1126271

**Published:** 2023-03-27

**Authors:** Yingying Liu, Renjun Gu, Murong Gao, Yangwa Wei, Yu Shi, Xu Wang, Yihuang Gu, Xin Gu, Hongru Zhang

**Affiliations:** ^1^ Institute for Immunology and School of Medicine, Tsinghua University, Beijing, China; ^2^ School of Chinese Medicine and School of Integrated Chinese and Western Medicine, Nanjing University of Chinese Medicine, Nanjing, China; ^3^ Beijing Rehabilitation Hospital Affiliated to Capital Medical University, Beijing, China; ^4^ Department of Hepatobiliary Surgery, Hainan Provincial People’s Hospital, Haikou, China; ^5^ Shanghai East Hospital, Tongji University School of Medicine, Shanghai, China; ^6^ School of Basic Medical Sciences, Fujian Medical University, Fuzhou, China; ^7^ School of Acupuncture and Tuina, School of Regimen and Rehabilitation, Nanjing University of Chinese Medicine, Nanjing, China; ^8^ The Second Hospital of Nanjing, Nanjing, China

**Keywords:** substance, energy, metabolism, neuroendocrine regulation, tumor cells

## Abstract

Cancer is the second most common cause of mortality in the world. One of the unresolved difficult pathological mechanism issues in malignant tumors is the imbalance of substance and energy metabolism of tumor cells. Cells maintain life through energy metabolism, and normal cells provide energy through mitochondrial oxidative phosphorylation to generate ATP, while tumor cells demonstrate different energy metabolism. Neuroendocrine control is crucial for tumor cells’ consumption of nutrients and energy. As a result, better combinatorial therapeutic approaches will be made possible by knowing the neuroendocrine regulating mechanism of how the neuroendocrine system can fuel cellular metabolism. Here, the basics of metabolic remodeling in tumor cells for nutrients and metabolites are presented, showing how the neuroendocrine system regulates substance and energy metabolic pathways to satisfy tumor cell proliferation and survival requirements. In this context, targeting neuroendocrine regulatory pathways in tumor cell metabolism can beneficially enhance or temper tumor cell metabolism and serve as promising alternatives to available treatments.

## 1 Introduction

Cancer is a disease that seriously threatens people’s life and health and is one of the leading causes of death each year, despite tremendous advances in detection and treatment in recent decades. According to statistics, there were about 23.6 million new cases of cancer worldwide and about 10 million people died from cancer in 2019 (1). Since 2000, the number of cancer cases and deaths as well as the crude incidence and mortality of cancer in China have gradually increased (2). Cancer is a heavy burden for both the patients themselves and the whole of society. At present, the global situation is still not optimistic. Therefore, it is crucial to find new regulated pathways of tumor cell death and investigate their therapeutic potential.

In the study of cancer biology, cancer metabolism represents one of the most important research directions. The synthesis, release, conversion, and utilization of energy in the whole metabolism are summarized under the term energy metabolism. Glucose is primarily converted to energy by cells. The primary energy source of normal cells is the aerobic oxidation of glucose, whereas the energy metabolism of tumor cells differs significantly from that of normal cells. The ability to reconfigure their metabolic network gives cancer cells the ability to adapt and ensure survival in the face of significant environmental change. During the 1920s, Warburg observed that the rate of glycolysis in tumor cells was significantly increased in tumor cells compared with normal cells. This phenomenon was later termed the Warburg effect, also known as aerobic glycolysis, which occurs in tumor cells even in the presence of sufficient oxygen (3). Despite its low production efficiency, glycolysis can rapidly produce ATP for tumor cells and also produce a variety of macromolecules to meet the material and energy requirements of tumor cells that proliferate rapidly. Although oxidative phosphorylation in mitochondria is an effective method for energy production, tumor cells prefer glycolysis as their method for energy production. Different tumor cells produce ATP in varying proportions from glycolysis and oxidative phosphorylation. In 2011, reprogramming of energy metabolism was named as one of the ten most important features of tumors (4). Reprogramming of energy metabolism not only provides energy and biomacromolecules for tumor cell growth and proliferation, but also supports tumor cell survival under stress conditions.

Surprisingly, a growing body of research has shown that neuroendocrine systems regulate a variety of molecular dynamics in substance and energy metabolism in tumors. To control numerous elements of energy intake, consumption, digestion, and absorption, the central nervous system (CNS) interacts with a number of peripheral organs and tissues (5). For example, food-induced changes in gastrointestinal tract tension can directly trigger vagal afferents, or indirectly activate taste receptors through chemical stimuli and trigger the production of gastrointestinal peptides (5). The released peptides, including ghrelin, gastric leptin, cholecystokinin, and peptide YY, or appetite-stimulating substances such as glucagon-like peptide 1 increase the feeling of satiety (5). Through circuits between the brainstem and hypothalamus, nutrient levels in the blood influence food intake (5). The circuits of homeostatic energy metabolism are called hypothalamic circuits (6). Neuropeptide Y (NPY) and dopamine pathways associated with sensory inputs of food such as smell and taste, and influenced by physiological states such as hunger and satiety, regulate food intake in the hypothalamus and extrahypothalamic nuclei (7, 8). In addition to food intake, the hypothalamic circuit controls other elements of energy homeostasis, such as fat metabolism (9), adipose tissue distribution (10), glucose metabolism (11), and insulin sensitivity (12). Energy expenditure, glucose and fat metabolism, and feeding behavior have been shown to change under stress (13). However, the neurobiology underlying these processes is constantly changing to meet the demands of energy supply in tumors. This review aims to highlight the molecular interface that neuroendocrine dynamics represent as an important general physiological condition for modulating tumor substance and energy metabolism and clinically determining cancer progression, and to provide a reference for basic research and clinical treatment of tumors by targeting neuroendocrine molecules.

## 2 Energy metabolism in tumor cell

Energy metabolism is one of the fundamental features of an organism’s life activities. Energy is needed for the growth and reproduction of cells. One of the reasons cancer why is so damaging to the body and so difficult to overcome is because of its ability to alter metabolic pathways, and give tumor cells a greater competitive advantage. Energy in cancer cells is provided mainly by adenosine triphosphate (ATP), with most of the ATP in the cells being generated by the breakdown of glucose, and a small amount by the breakdown of glutamine and fatty acid metabolism.

### 2.1 Glucose metabolism

**Figure 1:**
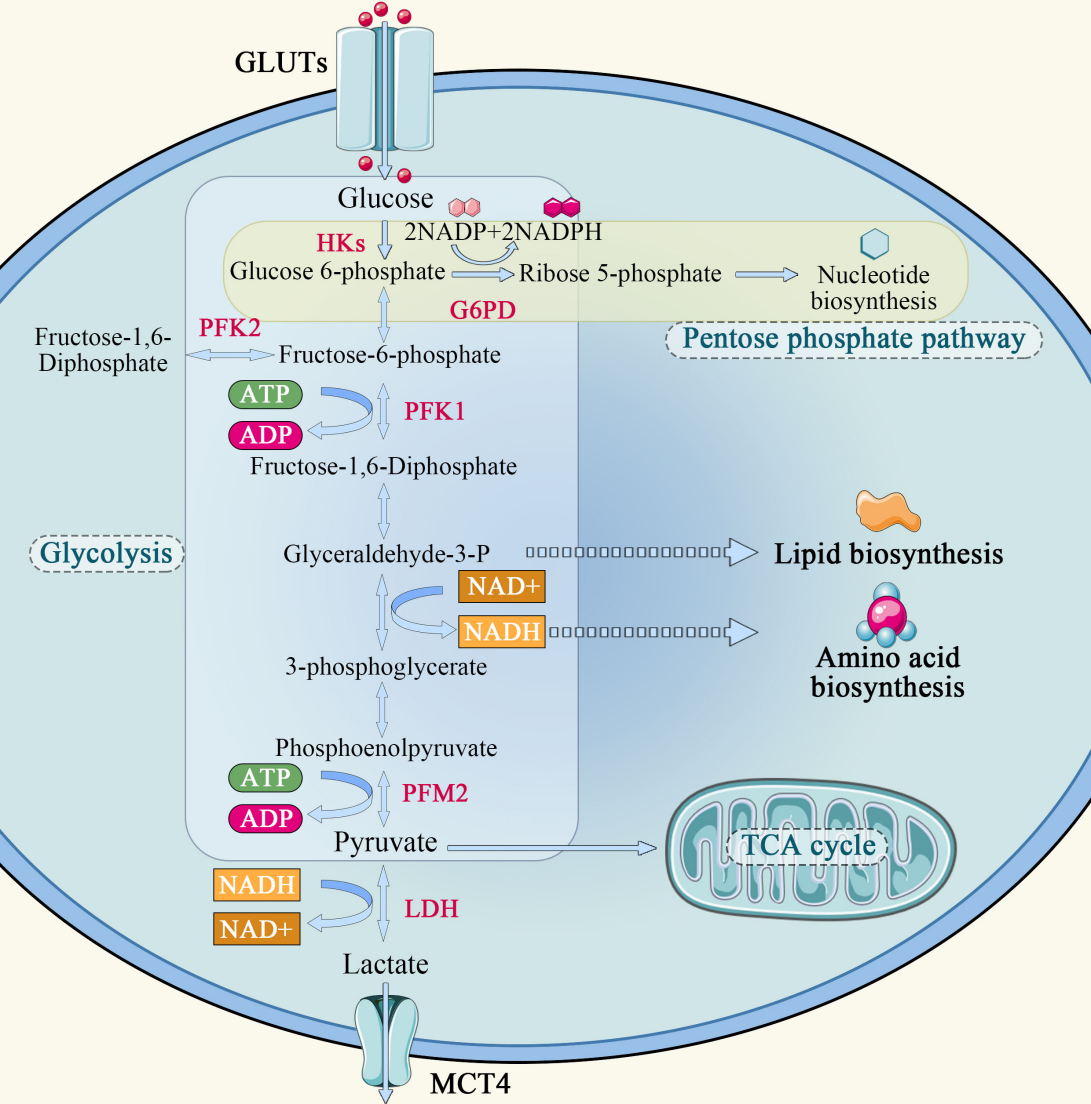
Schematic diagram of glucose metabolism in tumor cell.

In normal cells, the energy required for cellular metabolism is converted mainly from glycogen and other substances into 6-phosphate-glucose, and then enters the mitochondria *via* the glycolysis pathway, where it undergoes the tricarboxylic acid (TCA) cycle and oxidative phosphorylation, providing 70% of the energy required for its own metabolism. Glycolysis can only provide a small portion of the energy, which is about 20-30% of the metabolism of normal cells. The Warburg effect describes that how cancer cells tend to absorb glucose and convert it predominantly to lactate, even in the presence of oxygen, and refers to the abnormal glucose metabolism in cancer cells (**Figure 1**). The Warburg effect assumes that glycolysis is the main energy supply pathway for tumor cells, and that tumor cells rely on glycolysis for energy supply even when sufficient oxygen is available. Studies have shown that tumor cells transport extracellular glucose into the cell *via* glucose transporters distributed on the cell membrane and catabolize it to generate ATP using glycolytic enzymes such as hexokinase, phosphoglucose isomerase, and the product of the multistep metabolism of pyruvate. In the hypoxic region of the tumor, a large amount of lactate is formed from pyruvate by lactate dehydrogenase. Lactate is released to the outside of the cell through the only carboxyl transporter in the cell membrane and accumulates locally, creating an acidic environment for tumor growth. This microenvironment promotes tumor cell invasion into surrounding tissues (14). At the same time, researchers found that tumor cells in the oxygenated area could take up the lactic acid produced by cells in the hypoxic area and synthesize glucose through gluconeogenesis, which can be used by tumor cells in the hypoxic area to realize energy cycle (14). Lactic acid can also enter the bloodstream, reach the liver *via* gluconeogenesis, and eventually generate liver glycogen or blood glucose, resulting in a lactic acid cycle (15). In the oxygenated tumor oxygen region, tumor cells also have the same energy me of the TCA cycle as normal cells, i.e, the metabolite pyruvate enters the mitochondria through oxidative decarboxylation to form acetyl-CoA *via* transporters, and is oxidatively metabolized in the TCA cycle.

Aerobic glycolysis is a unique metabolic mode of tumor cells. The aerobic glucose metabolic pathway is actually a low-productivity metabolic pathway. One molecule of glucose is degraded to pyruvate *via* the glycolytic pathway, generating 2 molecules of ATP, whereas complete oxidation by oxidative phosphorylation in mitochondria generates 32 to 33 molecules of ATP. Tumor cells require a large amount of energy to proliferate rapidly, but they choose glycolysis, which is less productive. However, there is no obvious defect in the mitochondria of tumor cells. It has been found that mitochondria maintain complete functions in tumor cells, and the tumorigenic function of cancer cell lines *in vitro* and *in vivo* is reduced when mitochondrial DNA is specifically knocked down (4, 16).

Why do some tumor cells still prefer the less efficient pathway of glycolysis as their primary energy source, even though mitochondria are so efficient? First, the cytoplasm produces ATP 100 times faster than mitochondria, meaning the yield is low but the rate is high. As long as glucose supply is sufficient, the ATP produced by glycolysis per unit time is higher than that of oxidative phosphorylation (17). Second, the increase in glycolysis leads to the accumulation of metabolic intermediates that can generate the demand for tumor cell proliferation through other reactions. Finally, the massive accumulation of pyruvate during glycolysis generates lactate under the action of lactate dehydrogenase A (LDHA), which is transported outside the cell by monocarboxylic acid transporter 4 (MCT4), creating an acidic environment outside the cell that promotes tumor cell growth, invasion and metastasis.

In addition, tumor cells adapt to different survival conditions by altering their metabolism, a process known as metabolic plasticity. When using chemotherapeutic agents that target the proliferation phase of tumor cells, cancer stem cells (CSCs) can circumvent the killing effects of chemotherapeutic agents by regulating their own metabolic processes to keep them in a “resting state” with low energy metabolism. At the same time, CSCs also promote the metabolism of the pentose phosphate pathway and increase their own antioxidant capacity to adapt to different tumor microenvironments (TMEs) (18). Elgendy et al (19) also demonstrated through intermittent diet and drug administration that tumor cells have metabolic plasticity that can switch between glycolysis and oxidative phosphorylation to adapt to different survival challenges. Adenosine-activated protein kinase (AMPK) and HIF-1 are two important regulators of oxidative phosphorylation and glycolysis. To explain the Warburg effect in tumor metabolism, Sotgia et al (20) proposed that cancer-associated fibroblasts in the vicinity of the tumor are “induced” by cancer cells to switch energy metabolism to aerobic glycolysis and that these interstitial cells are “induced” by cancer cells. Metabolites of fibers can provide metabolic substrates for epithelial cancer cells as an energy source. In this model, interstitial cell glycolysis produces L-lactate and ketone bodies that provide raw materials for mitochondrial metabolism, and their transport to epithelial tumor cells with oxidative properties drives mitochondrial oxidative phosphorylation. This metabolic mode is also referred to as the “reverse Warburg effect” because mesenchymal cells, rather than tumor cells, take over aerobic glycolysis. At the same time, this also shows that tumor and tumor stromal cells belong to the same metabolic symbiosis.

### 2.2 Glutamine metabolism

Glutamine is the most abundant non-essential amino acid in human blood under normal conditions and accounts for about 50% of the free amino acids in the human body (21). In stressful situations, the body must supply glutamine to meet the demand, and glutamine is absorbed by the body and classified as a conditional non-essential amino acid. Under normal conditions, glutamine is synthesized and stored primarily in skeletal muscle, and some is also synthesized in adipose tissue, lung and liver, with skeletal muscle having the highest glutamine synthase activity. Glutamine taken up and stored by skeletal muscle is gradually released into the bloodstream and delivered to all parts of the body. The proliferation of lymphocytes and macrophages stimulated by antigens, and the renewal and maintenance of the intestinal mucosa require large amounts of glutamine. Therefore, the intestine and immune cells are important consumption organs for glutamine. 

**Figure 2:**
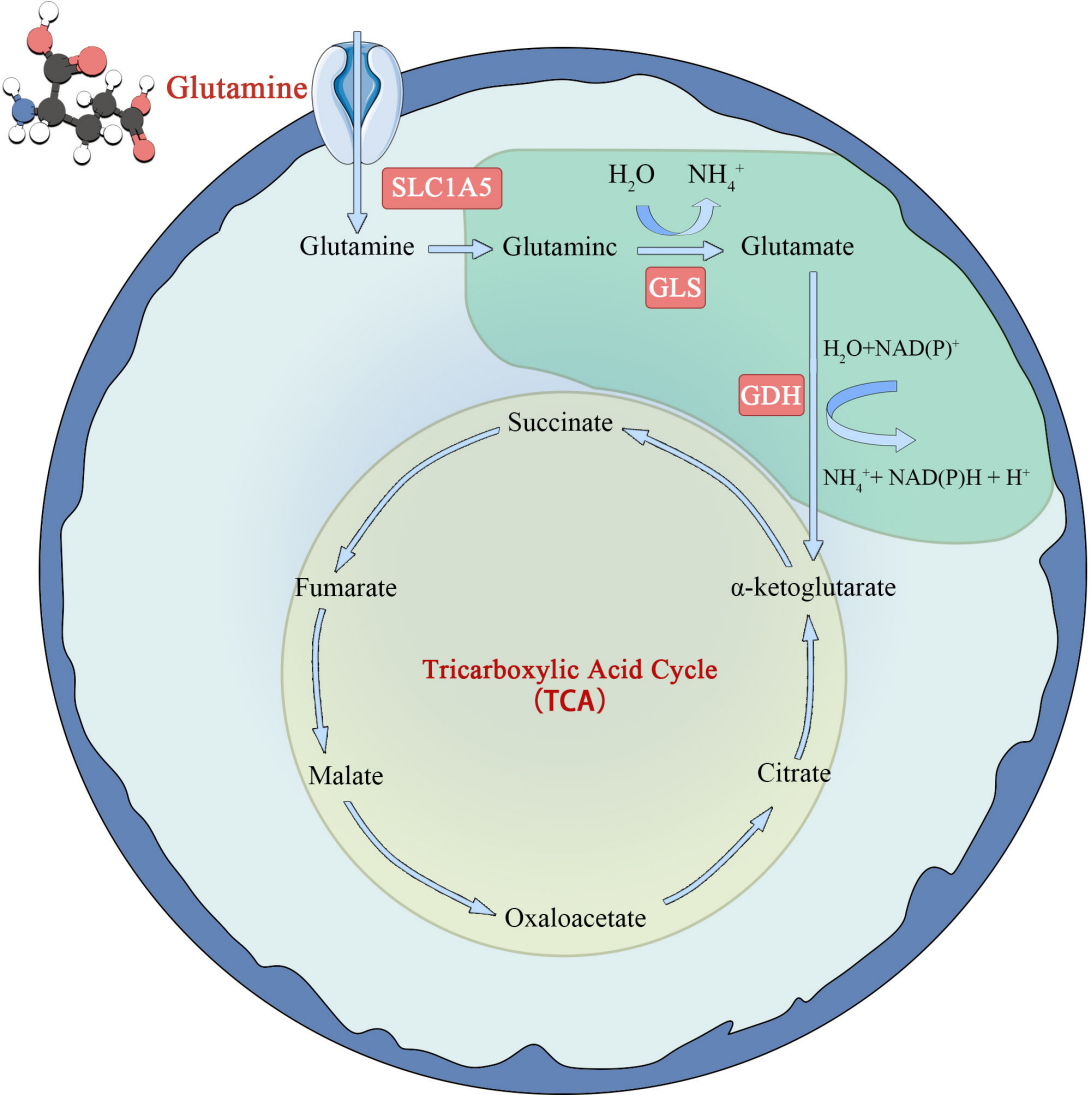
Schematic diagram of glutamine metabolism in tumor cell.

Glutamine metabolism is another characteristic of tumor cells (22) (**Figure 2**). Glutamate is synthesized from glutamate and ammonia under the catalysis of glutamine synthase (GS). However, in tumor cells or rapidly proliferating cells, the *de novo* synthesis of glutamine cannot meet the demand of cellular energy metabolism for glutamine, so it is converted to a conditionally essential amino acid. Glutamine enters the cell *via* the amino acid transporters SLClA5 and SLC7A5/SLC3A2, and is deaminated into glutamate in the mitochondria by glutaminase (GLS). Glutamate is formed under the action of glutamate dehydrogenase (GDH) or amino acid transaminase. Ketoglutarate (KG) is fed back into the TCA cycle and provides energy to cells through oxidative phosphorylation. The study found that tumor cells take up more glutamine and less glucose than immune cells in the TME. At the same time, it was observed that glutamine uptake and metabolism can significantly inhibit glucose metabolism. The specific mechanism is not clear (23), but it indicates that glutamine metabolism is very important for tumor cells. However, in later studies, glutamines was found to be an energy source only in some tumor cells and not in all tumor cells (24).

### 2.3 Fatty acid metabolism

In recent years, researchers have paid more attention to fatty acid metabolism in tumor cells because fatty acids are not only the main components of membrane formation, but also a source of energy supply and secondary messengers of signal transduction in rapidly proliferating tumor cells (25). In a state of energy stress, fatty acids in mitochondria produce acetyl-CoA through iodine oxidation, accompanied by the production of NADH and FADH, thereby supporting the cell’s biosynthetic pathway and producing ATP. In addition, phosphatidylinositol 3-kinase (PI3K) regulates several important signaling pathways. PI3K-AKT signaling pathway promotes glucose uptake and glycolysis by activating glucose transporter 1 (GLUT1) and hexokinase. PI3K-AKT signaling pathway can also enhance glutamine replenishment and lipid remodeling by activating glutamate pyruvate transaminase (26).

## 3 Neuroendocrine system

The endocrine/neuroendocrine system includes the endocrine organs like the pineal gland, adrenal gland, pituitary gland, thyroid gland, and parathyroid gland, as well as clusters of endocrine cells such as the pancreatic islets of Langerhans, bronchial neuroepithelial bodies, scattered epithelial endocrine cells (such as gastrointestinal endocrine cells), and neurons (27). In the literature, the terms “endocrine” and “neuroendocrine” are frequently used interchangeably, particularly when discussing neoplasms derived from these cells. In this review, we use the condensed classification system of the sympathetic nervous system (SNS) and hypothalamic–pituitary–adrenal (HPA) gland axis as the neuroendocrine system. The various distinct cell types composing this system produce and secrete a wide variety of amino acids, including glycine, glutamate, acetylcholine (ACh), and gamma-aminobutyric acid (GABA); biogenic amines including the neurotransmitters epinephrine (E) and norepinephrine (NE), and serotonin; neuropeptides including neuropeptide Y (NPY), vasoactive intestinal polypeptide (VIP), calcitonin gene-related peptide (CGRP), neurotensin, brainstem, and many others; steroid hormones including adrenocorticotropic hormone, growth hormone, hydrocortisone, and many others. The HPA axis is activated at the molecular level by the production of corticotrophin- releasing hormone and arginine vasopressin, both of which induce the release of adrenocorticotropin from the anterior pituitary gland as a crucial part of the hormonal response to dangerous stimuli. The following generation of glucocorticoids mediates the final output of the system (28, 29). Epinephrine and norepinephrine are produced by the sympathetic division of the SNS and the adrenal medulla, signaling physiological changes in response to a dangerous scenario (28, 29), which act either locally (paracrine function) or systemically *via* the vascular system (**Table 1**). Neuroendocrine regulation is the crucial element of the adaptive systems of organisms to regain homeostasis following environmental and psychosocial stresses. Both the SNS and HPA axis have been shown to modulate the substance and energy metabolism (28, 49), and other specific molecular processes implicated in these dynamics are also thought to influence the formation of tumors.

**Table 1:** Neuroendocrine mediators.

Neuroendocrine system	Neuroendocrine mediators	Reference
Amino acids	Glycine	(30)
	Glutamate	(31)
	Acetylcholine (Ach)	(32)
	Gamma-aminobutyric acid (GABA)	(33)
	Epinephrine (NE) and norepinephrine (E)	(34, 35)
	Serotonin	(36)
	Neuropeptide including neuropeptide Y (NPY)	(37)
Biogenic amines	Vasoactive intestinal polypeptide (VIP)	(38)
	Calcitonin gene related peptide (CGRP)	(39)
	Neurotensin	(40)
	Taurine	(41)
	6-alanine	(42)
	Hypocretin/Orexin (HO)	(43)
	Prooplomelanocortin (POMC)	(44)
Steroid hormones	Adreno corticotropic hormone	(45)
	Growth hormone	(46)
	Hydrocortisone	(47)
	Melanin-Concentrating Hormone (MCH)	(48)

## 4 Crosstalk between neuroendocrine regulation and tumor cell metabolism

**Figure 3:**
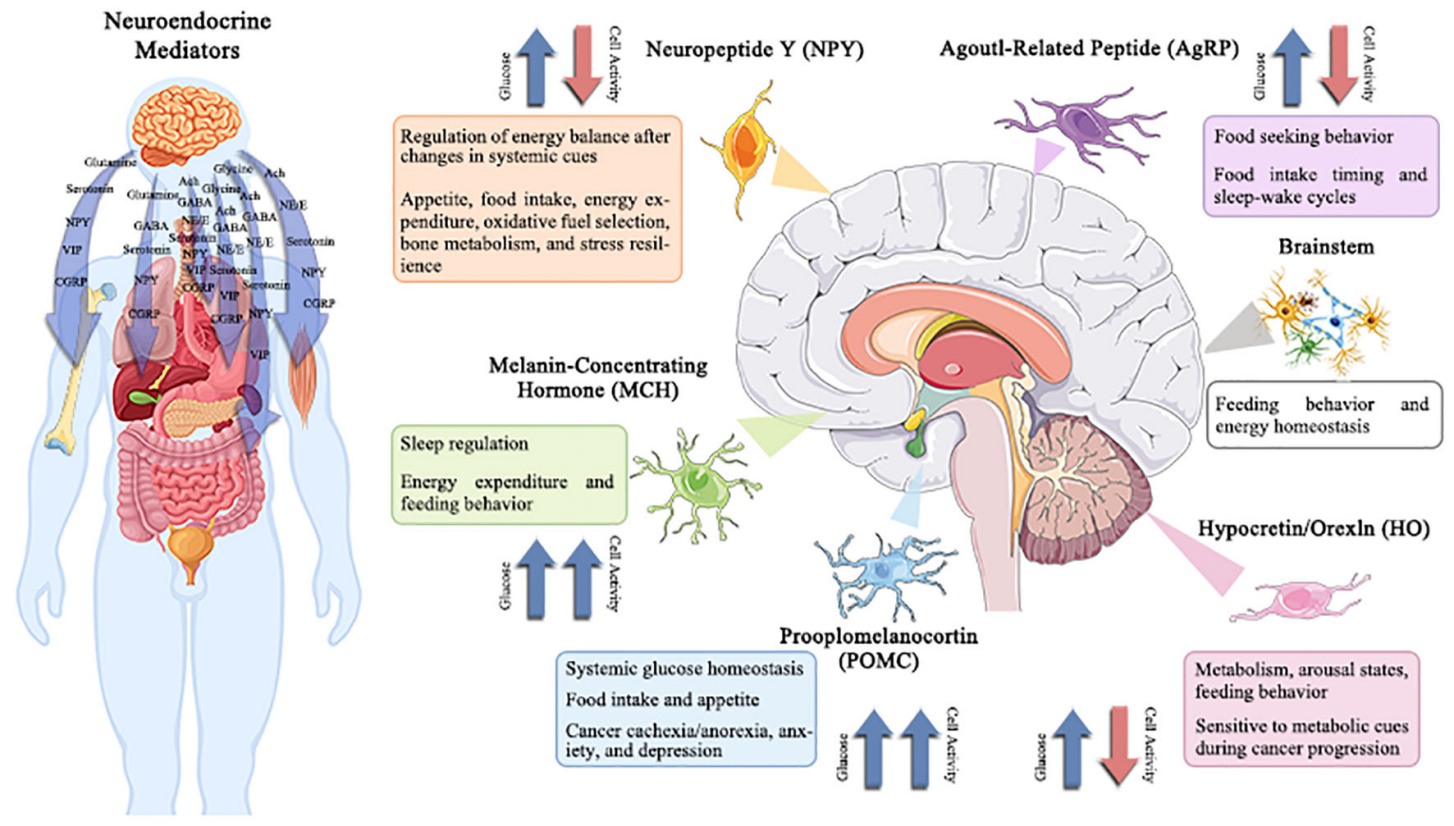
Potentially changed glucose-sensitive neuroendocrine mediator in the context of cancer-induced hyperglycemia.

Specific responses (inhibitory or excitatory) are displayed by specialized subsets of brainstem and hypothalamus neurons in response to variations in extracellular glucose concentrations (50). For proper control of systemic physiology, these two brain areas must work in close collaboration (51, 52). The lateral, arcuate, and ventromedial hypothalamic nuclei were identified to include hypothalamic glucose-sensing neurons in the 1960s (53); in contrast, the nucleus of the solitary tract, region postrema, and dorsal motor nucleus of the vagus was revealed to contain brainstem glucose-sensing neurons. Importantly, these neurons release mediators, which are essential for maintaining physiological homeostasis, controlling sleep-wake cycles, regulating food patterns, and other functions that are disturbed in cancer (**Figure 3**). Therefore, understanding the role of neurotransmitters play in the development of cancer provides a foundation for a suggested connection between psychosocial and physiological factors (54, 55). The functionality of migration of tumor cells has also been revealed to be significantly influenced by neurotransmitters and hormones (56). The section that follows will go through the impact of numerous traditional neurotransmitters and neuropeptides on the material and energy metabolism of tumors.

The hypothalamus and brainstem contain several neuroendocrine mediators that are sensitive to variations in extracellular glucose levels. These neuroendocrine mediators regulate a wide range of behavioral and physiological processes, including hepatic gluconeogenesis, energy balance, sleep/wake phases, eating behavior, and stress tolerance. Therefore, the effects of cancer-related alterations in glucose on central neuronal activity and subsequent physiology/behavior are anticipated to be extensive. Understanding and modifying these circuits may offer a unique strategy for treating co-morbidities linked with cancer, such as disturbed sleep, exhaustion, cachexia/anorexia, depression, and anxiety.

### 4.1 Epinephrine and norepinephrine

**Figure 4:**
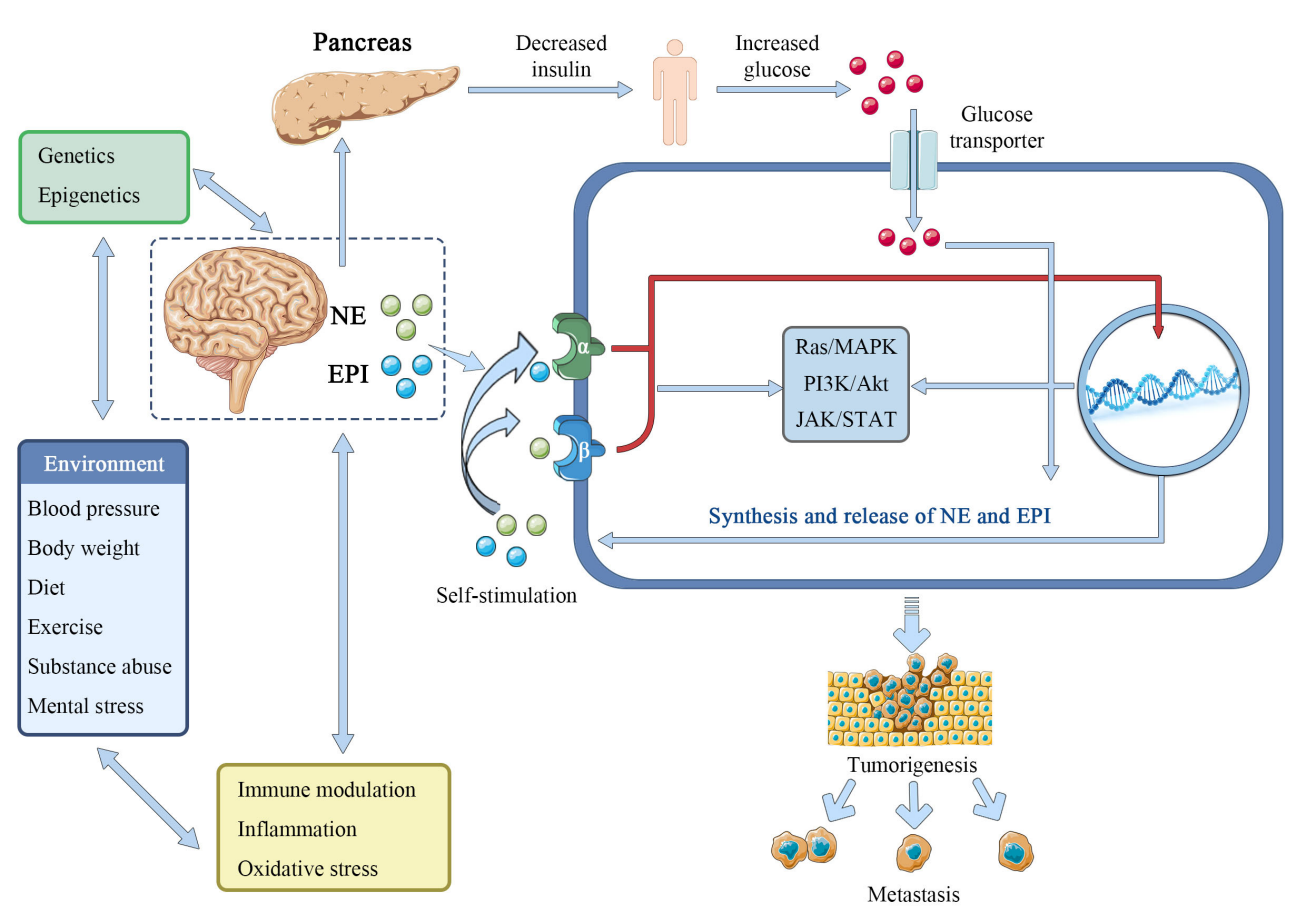
Epinephrine and norepinephrine contribute to tumorigenesis.

The catecholamines epinephrine (adrenaline) and norepinephrine (noradrenaline) are the best-known and most studied neurotransmitters, formed from the amino acid tyrosine and released mainly by sympathetic nerves and the adrenal medulla. The interactions between epinephrine and norepinephrine and the alpha (α)- and beta (β)-adrenergic receptors (ARs), which are G-protein-coupled 7-transmembrane receptors widely distributed in most tissues of mammals, mediate their actions. Epinephrine and norepinephrine, serve as stress hormones to respond to external stress or danger to the sympathetic and adrenal nervous systems (57, 58). Norepinephrine, in particular, plays a crucial role as a neurotransmitter in the brain and at the output of the sympathetic nervous system, which includes the network of peripheral nerves that controls the body’s organs. It was reported that there is a gender difference in responses to epinephrine. In men, but not in women, the release of free fatty acids (FFA) from lower body adipose tissue increased in response to epinephrine, whereas in both sexes the release of palmitate increased in the upper body. These results support some *in vitro* research and suggest that the differences in body fat distribution between males and females may be influenced by catecholamine activity (59). Furthermore, the biological properties of malignant tumors, such as cancer cell proliferation, invasion, metastasis, angiogenesis, resistance to apoptosis, and stromal compartments in the tumor microenvironment, are strongly influenced by epinephrine and norepinephrine (60). Isoproterenol, an α-adrenergic agonist, can imitate the tumor growth and angiogenesis brought on by prolonged stress, while propranolol, an α-adrenergic antagonist, can prevent this (61). Importantly, epinephrine and norepinephrine have been considered to be one of the main regulators in the metabolism of tumor cells. In breast cancer survivors, epinephrine, cortisol, and lactate responses appeared to be attenuated compared with controls, while glucose and responses showed larger magnitude changes. The adrenergic system regulates energy balance in part by promoting thermogenesis and the release of lipids from brown or white adipose tissues (62, 63), and human fat cells are equipped with adrenergic receptors (adrenoceptors) β_1_ (ADRB1), β_2_ (ADRB2) and β_3_ (ADRB3). Beta-adrenergic genes have already been linked to a variety of cancers, including their interactions with environmental or other risk factors (64–67). Adrenoceptor polymorphisms and the dopamine beta-hydroxylase enzyme, which produces norepinephrine, can modify insulin resistance and change glucose signaling (68–71), which may have an impact on the Warburg effect. Norepinephrine can activate the metabolism of endothelial cells to block oxidative phosphorylation and activate an angiogenic switch that promotes the growth of cancer (72, 73). In pancreatic cancer, catecholamines promote neurotrophins to be secret by β-ARs, which in turn raises norepinephrine levels and aids tumor growth (74). Chronic stress-induced epinephrine promotes the development of breast cancer stem-like traits by rewiring the metabolism in a lactate dehydrogenase A (LDHA) dependent manner (75). Catecholamines norepinephrine and epinephrine have been demonstrated to play a role in metabolic reprogramming and epithelial-to-mesenchymal transition in liver and colorectal cancers (76, 77). PCK1 regulates glucose metabolism and neuroendocrine differentiation through the activation of LIF/ZBTB46 signaling in castration-resistant prostate cancer (78). Together, these and other numerous studies provide compelling evidence that epinephrine and norepinephrine play an important role in the metabolism of substances and energy, which promotes the growth and spread of tumors in multiple of cancer types (**Figure 4**).

Epinephrine (EPI) and norepinephrine (NE) interact with environmentally-regulated factors like obesity, hypertension, unhealthy dietary components, physical inactivity, substance abuse, and mental or emotional stress to promote the Warburg effect by facilitating glucose. These interactions are in addition to the direct interaction of elevated central catecholamine release or peripheral sympathetic-adrenomedullary signaling with epigenetic and genetic risk factors including mutagenesis, and perhaps by increasing insulin resistance. Additionally, it is suggested in this research that many cancer cells produce and release catecholamine molecules to autocrinely activate their own α-ARs and β-ARs. To encourage cancer and metastasis, EPI and NE may potentially interact with oxidative stress, systemic inflammation, and immunological function.

### 4.2 Gamma-aminobutyric acid (γ-GABA)

γ-GABA is the adult mammalian brain’s major inhibitory neurotransmitter for CNS. The ionotropic GABA_A_ and GABA_C_ receptors as well as the metabotropic GABA_B_ receptor are three distinct types of GABA (A, B, and C) receptors. Numerous tumor tissues have been found to contain GABA receptors, which control the migration and proliferation of tumor cells (79, 80). The GABAergic system and the growth of tumors appear to be closely associated, according to recent research using human cancer cell lines, animal models, and human tissues. In general, stimulation of GABA receptors slows migration (81) and suppresses tumor cell proliferation (82). These findings imply that the GABAergic system contributes significantly to cell pathology, and it is possible that GABA plays a substantial role in the prognosis of cancer patients. Some cancers has been shown to have higher GABA levels, such as breast cancer (80), ovarian cancer (80), gliomas (83), gastric cancer (84), colon cancer (85), and prostate cancer (86). Typically, GABA inhibits cancer cell growth through the GABA_B_ receptor, but stimulates cancer cell growth through the GABA_A_ receptor pathway (87). The GABAergic system and the growth of tumors appear to be closely related, according to recent research using human cancer cell lines, animal models, and human tissues. Recently, it was demonstrated that two independent 13C-labeled substrates, [1,6-13C2] glucose and [2-13C] acetate, which are metabolized in neurons and glia differently, may be used to evaluate the TCA cycle and neurotransmitter cycle fluxes of glutamatergic and GABAergic neurons *in vivo* separately (88). Using this technique in adult rats under halothane anesthesia, it was found that cortical glutamatergic and GABAergic neurons contribute 80% and 20%, respectively, of neuronal glucose oxidation and neuronal/glial cycling (88). The γ-GABA abnormality is present in many diseases and can be served as potential target. It was reported that abnormalities in Glu/GABA-Gln are present in rat dyskinetic syndrome, and the amino acid neurotransmitter imbalance was improved by “Tiapride,” which also increased the expression of GS and EAAT2 protein, decreased Glu levels, increased γ-GABA levels, and increased γ-GABA levels (89). Additionally, treatment with 10 mM γ-GABA considerably slowed down the loss of malate and titratable acidity and increased the levels of succinate and oxalate. Fruit treated with GABA had higher cytosolic activities of nicotinamide adenine dinucleotide-dependent malate dehydrogenase (cyNAD-MDH) and phosphoenolpyruvate carboxylase (PEPC) than control fruit, whereas administration of 10 mM GABA significantly reduced the loss of malate and titratable acidity and raised the concentrations of succinate and oxalate. GABA-treated fruit had larger cytosolic activities of nicotinamide adenine dinucleotide-dependent malate dehydrogenase (cyNAD-MDH) and phosphoenolpyruvate carboxylase (PEPC) than control fruit, although cyNADP-ME and phosphoenolpyruvate carboxykinase activities were lower. Notably, GABA administration drastically decreased ethylene production while also downregulating the expression of MdACS, MdACO, and MdERF. GABA therapy also boosted the accumulation of GABA and improved the function of the GABA shunt (89). The GABA_A_ receptor agonist muscimol promotes gastric cancer cell growth by triggering mitogen-activated protein kinases (MAPK). Similar to this, GABA promotes the formation of pancreatic cancer by increasing intracellular Ca^2+^ levels and the MAPK/ERK cascade by overexpressing GABRP, a subunit of GABA_A_ (90). Contrarily, activation of GABA_B_ receptors successfully prevents DNA synthesis and cell migration by inhibiting isoproterenol-induced cAMP, p-CREB, cAMP response element-luciferase activity, and ERK1/2 phosphorylation (91). The GABA or GABA_B_ agonist baclofen has been demonstrated to promote Epidermal growth factor receptor (EGFR) transactivation, which has been connected to the propensity of prostate cancer cells to invade (92). According to these findings, various GABA activation-induced effects on cancer development and migration may vary on the kind of cancer or GABA receptor. Contrary to the mechanism described above, our most recent research showed that the GABA_A_ receptor subunit promotes the growth of pancreatic cancer by altering KCNN4-mediated Ca^2+^ in a GABA-independent manner (93). Besides, it is intriguing that GABA is present in the tumor microenvironment, which suggests that it may be able to control inflammation by concentrating on immune cells that have invaded the tumor (93). In summary, these advances remind that nutrition has evidently metabolic consequences that may change the incidence and progression of cancer, reinforcing the metabolic cancer model.

### 4.3 Glutamate

In brain tissue, glutamate is widely distributed and has the highest concentration all amino acids. Over the past 50 years, numerous studies have been conducted on the functions of glutamate in the brain, revealing a wealth of information about glutamate. Early research by Krebs indicated that glutamate has an important metabolic function in the brain (94). Waelsch and colleagues made the first observation about the complicated compartmentation of glutamate metabolism in the brain (95). Neurotransmission in both cell types has the highest energetic cost, which increases with cortical activity. Interpretation of functional imaging results is significantly influenced by the contribution of GABAergic neurons and inhibition to cortical energy metabolism (88). Using NAD or NADP as cofactors, glutamate dehydrogenase (GDH) catalyzes the oxidative deamination of glutamate to α-ketoglutarate. GDH is found in primarily in astrocytes in the mammalian brain, where it is likely involved in the metabolism of the transmitter glutamate. Thus, while GTP primarily controls housekeeping GDH, the availability of ADP or L-leucine has a significant impact on GDH activity in neural tissue. GDH specific to neural tissue is likely to be activated under circumstances that promote hydrolysis of ATP to ADP (e.g., during intense glutamatergic transmission), increasing glutamate flux through this pathway (96). In synaptosomes and cultured neurons that do not produce GDH, the rate of oxidative glutamine metabolism was significantly lowered when glucose was restricted. In contrast, the absence of GDH expression had no impact on glutamine metabolism when glucose was present. In brain mitochondria from GDH KO mice, respiration powered by glutamate was significantly lower, and synaptosomes were unable to increase their respiration in response to increased energy demand. The importance of GDH for neurons, especially during times of high energy demand, is highlighted by its role in the metabolism of glutamine and the capacity for respiration. This may be due to the significant allosteric activation of GDH by ADP (97). Using 13C, astroglia plays a role in energy metabolism of human brain. The primary pathway for the neurotransmitter glutamate repletion has been identified, and nuclear magnetic resonance spectroscopy has been used to study astrocytic oxidative metabolism (98). The pathophysiology of hyperammonemia and hepatic encephalopathy appears to be heavily influenced by abnormalities in glutamate metabolism and glutamatergic neurotransmission. The pathogenesis of hepatic encephalopathy and other hyperammonemia conditions involves an abnormality in astroglial glutamate uptake caused by ammonia (99). Additionally, in the absence of *ad hoc* activity-related metabolic restrictions, the glutamate-glutamine cycle does not control the relative energy requirements of neurons and astrocytes, and as a result, their intake of glucose and the exchange of lactate (100). Glutamate-induced Ca^2+^ loads cause mitochondria to sequester Ca^2+^, which then uncouples respiration and results in metabolic acidosis. The acidification brought on by glutamate is a sign of metabolic stress and may suggest that mitochondria are crucial in the process of glutamate-induced neuronal death (101).

### 4.4 Dopamine

Dopamine served as a minor intermediary in the synthesis of noradrenaline in 1957. Today, it is a significant neurotransmitter in the brain. It was reported that dopamine plays a key role in modulating learning and motivation. Excitatory and inhibitory synaptic transmission are altered by dopamine. While the nature of neuromodulation of inhibitory transmission is still under discussion, it appears that activation of the dopamine 1 (D1) receptor specifically promotes N-methyl-D-aspartic acid receptor (NMDA) but not α-amino-3-hydroxy-5-methyl-4-isoxazole-propionicaci (AMPA) synaptic transmission in the cortex and striatum. Because of their dependence on voltage, NMDA currents are less active when the postsynaptic cell is not firing than they are when it is depolarized. Large networks of pyramidal neurons may be induced to enter bistable states resembling working memory, according to experimental and theoretical data (102). The capacity of the striatum to store dopamine as assessed by L-[^18^F]-fluorodopa uptake was normal, but dopamine (D2) receptor binding was decreased in huntington’s disease compared with normal subjects (103). In addition, glutathione is a critical neuroprotectant for midbrain neurons in conditions when energy metabolism is compromised and show that an oxidative challenge occurs during suppression of energy metabolism by malonate (104). Parkinson’s disease (PD) neurons had damaged PI3K/Akt, mTOR, eIF4/p70S6K, and Hif-1 pathways, which are part of a network regulating energy metabolism and cell survival in response to growth factors, oxidative stress, and nutrient deprivation. The primary hubs of this network, which is important for longevity and may be a target for therapeutic intervention along with the stimulation of mitochondrial biogenesis, are PI3K/Akt and mTOR signaling (105). Recently, a distinct metabolomic profile linked to parkin dysfunction and demonstrate the value of combining metabolomics with an iPSC-derived dopaminergic neuronal model of parkinson’s disease to gain fresh understanding of the pathogenesis of the disease (105). The striatum and prefrontal cortex of the spontaneously hypertensive rat model of attention deficit hyperactivity disorder (ADHD) show impaired energy metabolism and disturbed dopamine and glutamate signaling (106). Several metabolic abnormalities, including insulin resistance, abdominal obesity, dyslipidemia, and hypertension, make up the metabolic syndrome. Its pathogenesis may be influenced by faulty dopamine D2 receptor (D2R) signaling, according to earlier studies. D2R activation simultaneously improves various metabolic traits in obese women (107). The failure of dopamine and glutamate’s connection in controlling energy metabolism results in neuronal death (108). Midbrain dopaminergic cells with Lesch-Nyhan disease have limited developmental potential and impaired energy metabolism (109). Catecholamine toxicity may result from interactions with the mitochondrial electron transport system as well as from the induction of an oxidative stress state, and this was further supported by the fact that ADP was able to reverse the dose-dependent inhibition of NADH dehydrogenase activity caused by dopamine (110).

### 4.5 Serotonin

Serotonin (5-hydroxytryptamine [5-HT]) is a monoamine that has a variety of effects on the peripheral organs as well as the CNS. In the brain, 5-HT is a neurotransmitter that regulates mood, sleep, behavior, appetite, and other functions (111). Serotonin is also an important regulator of the inputs to the energy balance, including energy intake and energy expenditure. Serotonin in the CNS plays a complex and intricate role in appetite and subsequent nutrient intake (112). Receptor agonists for the treatment of obesity have been approved due to serotonin’s inhibition of appetite (113). The rate-limiting enzyme tryptophan hydroxylase (TPH) transforms the amino acid tryptophan into 5-hydroxtryptophan (5-HTP), which is then converted to 5-HT by aromatic acid decarboxylase. TPH2 is expressed in the CNS and peripheral neuronal tissues, whereas TPH1 is present in peripheral nonneuronal tissues. These two isoforms of TPH were discovered to be expressed in a mutually exclusive pattern in the early 2000s (114). Since 2010, scientists have become more aware of how peripheral serotonin controls systemic energy metabolism. The enterochromaffin cells of the gut produce the majority of the 5-HT present in the body. However, 5-HT is also generated by many metabolic organs and has been shown to have biological effects that are endocrine, paracrine, and autocrine in nature. 5-HT promotes proliferation and mass enlargement of pancreatic β-cells. 5-HT encourages lipogenesis and prevents adaptive thermogenesis in adipose tissues. 5-HT activates hepatic stellate cells and causes lipogenesis and gluconeogenesis in the liver (115). It was reported that dairy cows in late lactation treated with 5-HTP had improved energy metabolism, reduced urinary calcium loss, and increased milk calcium secretion. To ascertain any advantages for post-partum calcium and glucose metabolism, additional research should focus on the effects of increased serotonin during the transition period. Chronic acetyl-l-carnitine administration reduced the conversion of glucose to lactate, elevated energy metabolite levels, and changed the levels of monoamine neurotransmitters in the mouse brain (116). Recent genetic studies suggest that leptin signaling physiological processes, most notably leptin’s control over appetite and the accumulation of bone mass, are primarily involved in the inhibition of serotonin synthesis and released by brainstem neurons (117). Collectively, 5-HT plays an emerging role in regulating metabolism in cancer cells.

### 4.6 Neuropeptides

Neuropeptide Y (NPY) is one of the most prevalent neuropeptides in the brain, with 36 amino acids (118, 119). In order to regulate hunger and energy balance, agouti-related protein neurons (AgRP) in the CNS emit NPY, which was first identified as a powerful neuropeptide that stimulates appetite (120–122). The central regulatory effects of NPY on circadian rhythm, the cardiovascular system, stress, and anxiety were gradually demonstrated as this peptide’s role in the body’s regulation of these processes became better understood (123). Mammals have five different types of NPY receptors (Y1, Y2, Y4, Y5, and Y6), which are found throughout the CNS (124, 125) and linked to various stages of oncogenesis, allowing NPY to exercise its biological effects. When Y2-R is activated, it appears to encourage angiogenesis, whereas Y1-R appears to be involved in the regulation of cancer cell growth. Furthermore, a thorough investigation of the NPY receptor revealed that it is expressed in peripheral tissues such as adipose tissue, the pancreas, and bone (126, 127). As a result, the peripheral effect of NPY has drawn a lot of attention. For instance, activating the NPY receptor in the pancreas can lower hyperglycemia and β-cell apoptosis (128). Adipocyte proliferation and adipogenesis are promoted by NPY in adipose tissue (127). This suggests that in addition to being secreted in the brain by peripheral tissues, NPY also plays significant regulatory roles in the endocrine system (129, 130). In addition to these conventional functions, neuropeptides have been shown to promote tumor growth (131, 132). Numerous neuropeptides, including SP and NPY (133) have been thoroughly investigated in malignancies. Neuropeptide receptors are often GPCRs, which is a superfamily of receptors. For instance, the neurokinin-1 (NK-1) receptor, which is connected to the Gq family of G proteins, is primarily responsible for the pharmacological activity of SP. Upon activation, the NK-1 receptor produces the second messenger’s inositol 1,4,5-triphosphate (IP3) and diacylglycerol (DAG) (134). Through its effects on energy homeostasis, the NPY system has complex and significant implications for the development of cancer. Botox particularly, but not only, suppresses NPY in cancer using *in vitro* models and tissues from a prior human chemical denervation investigation. NPY nerve quantification is an independent predictor of prostate cancer-specific mortality. Last but not least, radiation-induced apoptosis is reduced when prostate cancer cells are cocultured with dorsal root ganglia/nerves, and NPY-positive nerves are increased in the prostates of patients who failed radiation therapy, suggesting that NPY nerves may be involved in radiation therapy resistance (135). In summary, understanding the role of NPY in whole-body energy balance could provide insight on mechanisms underlying the pathogenesis of cancer.

## 5 Therapeutic implications of the interaction between energy metabolism and neuroendocrine regulation

In the past ten years, numerous advancements have been made to reprogram the highly dynamic and heterogeneous energy metabolism of cancer cells. Cancer was first recognized as a disease with altered metabolism one hundred years ago. Migration, invasion, and metastasis are significantly influenced by metabolic alterations in the tumor cell. Despite a lengthy study history, the intricate connections between tumor metabolism, tumor development, and immunosuppression continue to be fascinating fields of study. For the creation of anti-cancer medications, modifications in tumor cell metabolism, such as increased glycolysis, glutaminolysis, and fatty acid metabolism, constitute appealing targets (136). Targeting the metabolism of tumor cells, however, is a strategy that can indirectly affect stromal components like fibroblasts or immune cells in addition to directly killing tumor cells.

In many distinct forms of human malignancies, the neurotransmitters variably control a wide range of activities of cancer cells, endothelial cells, and immune cells. The increasing involvement of the neurotransmitter system in tumor biology and the tumor microenvironment is now better understood, creating new potential for the development of cancer-targeted treatments. Many traditional neurotransmitter-related medications, including β-AR antagonists, serotonin receptor antagonists, AChR antagonists, and DR agonists, may have clinical consequences in the treatment of cancer and be interesting candidates for combination drug therapy. Further research should be done on surgical or chemical denervation and targeting neurotrophic signaling to avoid neoneurogenesis as a cancer treatment option. It is interesting to note that recent research suggests that a number of neurotransmitters, including 5-HT, dopamine, NE, and histamine, may act as substrates for protein posttranslational modification, such as the well-known crotonylation of histones (137). Selective serotonin reuptake inhibitors (SSRIs) or other small compounds that act on biogenic amines or transglutaminase may therefore prove to be a cutting-edge treatment for cancer. However, additional research is necessary to solidify the use of these medications in the arsenal of cancer therapy and to prevent side effects.

Notably, several neurotransmitters and their analogs, antagonists, and agonists for their receptors have therapeutic benefits and are used as medications for various illnesses, including cancer. Around 2008, a team of French physicians published a paper suggesting the use of propranolol, which inhibits β-ARs (i.e., receptors that EPI and NE activate), to reduce or eradicate benign tumors known as infantile hemangiomas in newborns (138). Since then, numerous academic publications have been written about this subject, and therapeutically, propranolol has taken the place of other treatments for malignancies (139). Meanwhile, studies using *in vitro* preparations, *in vivo* rodent models, and retrospective epidemiological studies of human subjects have suggested that propranolol is therapeutic in a variety of cancer types (counteracting both tumorigenesis and metastasis, including when combined with other pharmacological agents) (140, 141). According to a recent, well-known retrospective study, women with ovarian cancer who used non-selective beta-blockers (like propranolol) had a median overall survival of 94.9 months, compared with 42 months for non-users (142). Propranolol guards against disease recurrence in people with thick cutaneous melanoma, according to a prospective human subjects study (143). Numerous preclinical and clinical studies suggest that propranolol may have therapeutic benefits for angiosarcoma, poor prognosis or refractory cancer (144, 145). Additionally, propranolol is the subject of numerous ongoing clinical trials for a range of different neoplasms. Prazosin (which blocks the alpha1 adrenoceptor) and other medications other than propranolol that also block adrenoceptors are therapeutic in rat models, and additional research has suggested that NE itself promotes cancer (146, 147). In addition, to modify tumorigenesis and metastasis, the molecules serotonin, acetylcholine, and melatonin may act centrally or interact with the sympathetic-adrenomedullary system in the periphery (148, 149). Propranolol, a non-selective beta-adrenoceptor (beta1 and beta2) blocking medication, is being studied more and more for its potential to prevent or treat a variety of human cancers (150). However, in a specific situation, cancer cells need not produce their own NE/EPI or release it in an autocrine manner in order to be responsive to propranolol treatment, as this medication or those in its family (carvedilol, nebivolol) may lower blood sugar by altering pancreatic insulin release or improving insulin sensitivity (151). Propranolol and related beta-blockers, including in breast cancer cells, may enhance glycemic control through modulation of GLUT4 glucose transporter expression and hexokinase-2 (152, 153). In this case, propranolol might also inhibit beta-adrenoceptors on the cancer cell’s extracellular surfaces, which would be responding to NE/EPI from non-autocrine sources like the adrenal glands. Additionally, propranolol (or related medications) can still inhibit beta-adrenoceptors on the surface of cancer cells, dampening intracellular molecular pathways linked to cancer, even in the absence of the Warburg effect in cancer cells. Additionally, propranolol or closely related medications may still be able to lower blood sugar levels *via* the pancreas or improve insulin sensitivity even in cases where cancer cells exhibit the Warburg effect but lack adrenoceptors.

Differences between normal and mutant oncogenic enzymes and cancer cells’ addiction to nutrients to support uncontrolled cell growth programs imposed by cancer genes are the therapeutic windows for addressing cancer cell metabolism. Therefore, the intricate regulatory networks involving cancer genes and metabolic pathways must be identified for particular cancer types in order for somatic genetic changes in tumors to strategically direct the targeting of cancer cell metabolism. It is hoped that during the next ten years, new medicines will emerge from the fundamental sciences of metabolism, with the increase in knowledge and interest in cancer metabolism.

## 6 Concluding remarks

Cancer cells develop the capacity to remodel their metabolic network, enabling them to adjust and maintain their survival in the face of drastic environmental changes. The rate of glycolysis in tumor cells was significantly increased in tumor cells compared to normal cells, which was termed the Warburg effect, also known as aerobic glycolysis, which occurs in tumor cells even in the presence of sufficient oxygen. Glutamine metabolism is another characteristic of tumor cells. In addition, fatty acids are not only the main components of membrane construction, but also a source of energy supply and secondary messengers of signal transduction in rapidly proliferating tumor cells. Neuroendocrine control is crucial for tumor cells’ consumption of nutrients and energy. There is a crosstalk between neuroendocrine regulation and tumor cell metabolism. Numerous traditional neurotransmitters and neuropeptides including epinephrine and norepinephrine, γ-GABA, glutamate, dopamine, serotonin and neuropeptides have an impact on the material and energy metabolism of tumors. As a result of understanding the neuroendocrine regulatory mechanism of how the neuroendocrine system can fuel cellular metabolism, better combinatorial treatment methods will be possible. Innovative anti-cancer medicines may be based on research on tumor metabolism and neuroendocrine influences on tumors. The creation of medications that directly affect the altered tumor metabolism at the neuroendocrine level may prove to be a ground-breaking oncology treatment. These novel understandings of key catabolic pathways in cancer provide a focus for further research in this field and may aid in the development of effective therapeutic strategies.

## Author contributions

YG guided the proof of the manuscript, and offer the financial support for the project leading to this publication. All authors contributed to the article and approved the submitted version.
